# Design of a high-efficient SiC-based interleaved voltage source converter

**DOI:** 10.1016/j.heliyon.2024.e33227

**Published:** 2024-06-27

**Authors:** Adolfo Dannier, Gianluca Brando, Marino Coppola, Ciro Attaianese

**Affiliations:** Dept. of Electrical Engineering & IT, Univ. of Naples “FEDERICO II”, Via Claudio, 21, Napoli, 80125, Italy

**Keywords:** Interleaved voltage source converter (IVSC), Converter topologies, High-efficient design, Electric motor drives

## Abstract

The paper deals with some peculiar aspects of the design of interleaved voltage source converter (IVSC) topology in high-power applications such as renewable energy systems and electric vehicles. The IVSC offers advantages like power sharing among multiple modules, improved power quality, redundancy, and fault tolerance. The design of an IVSC mainly involves the selection of the number of deployed converters and the choice of suitable semiconductor devices with the aim to reach a desired overall efficiency for an assigned operating condition. The paper develops an approach to characterize through a straightforward procedure the dependence of the IVSC' power losses on the number of the interlaced converters, on the employed semiconductor devices used and on a properly conceived set of key parameters which define the system operating conditions. The proposed methodology allows to highlight the system performance' dependence on the chosen power devices and it is showcased in this work by considering silicon carbide (SIC) devices with different current ratings. The main goal of the paper is to formulate a smart design procedure able to guide the configuration of an IVSC towards an optimal choice with respect to the power module rating, the number of interleaved levels, the system efficiency, and the output current waveforms. The proposed procedure has been experimentally validated.

## Highlights

• Interleaved Voltage Source Converters increase the overall power rating without changing the power devices.
• Interleaved Voltage Source Converters allow effective current sharing between the power devices.
• Interleaved Voltage Source Converters are characterized by both the power devices and the interleaved level.
• Interleaved Voltage Source Converters can be designed through a proper formalization of the efficiency behavior.

## 1 Introduction

The Interleaved Voltage Source Converter (IVSC) is a power electronic converter topology commonly used in high-power applications such as renewable energy systems, motor drives, and grid-connected systems [1], [2], [3], [4]. It offers several advantages over other converter topologies. In particular, since the IVSC divides the total power rating between multiple converter modules connected in parallel, it allows both greater overall power and a reduction of the rated current of individual power devices to be obtained at the same time. In this way, the use of smaller and less expensive components is enabled, [5], achieving a greater redundancy and fault tolerance [6], [7]. The IVSC employs interleaved pulse-width modulation (PWM) techniques, where the switching instants of each module are knowingly staggered. This significantly reduces the low-order harmonics in the waveforms of the output voltage and current, resulting in improved power quality, reduced total harmonic distortion (THD), and lower impact on the grid and associated equipments [8]. With reference to the efficiency, the parallel operation of multiple modules in an IVSC reduces the current rating of individual devices, leading to reduced conduction losses [9], [10]. Additionally, [11], the interleaved PWM operation spreads the switching losses over multiple devices, reducing overall losses. Consequently, IVSCs can achieve high conversion efficiency, contributing to get energy savings and reduced operating costs. IVSCs have gained significant attention and are being increasingly utilized in electric vehicle (EV) applications [12], [13], due to the requirement for high current to ensure adequate traction power, given the limited DC-Link voltage. In this context, the sharing of the total current among multiple components is undoubtedly a significant advantage for enhancing overall system efficiency and management. The utilization of IVSCs also brings about additional indirect benefits: the reduced harmonic distortions in the output voltage and current waveforms ensure low electromagnetic interference (EMI) and minimize stress on other components of the charging infrastructure; IVSCs are inherently scalable, making them suitable for different power levels and charging infrastructure requirements. EV charging stations can utilize multiple IVSC modules to accommodate various charging power levels and future expansion needs. The parallel operation of multiple IVSC modules provides redundancy and fault tolerance. In EV charging applications, this means that if one module fails, the remaining modules can continue operation, reducing downtime and ensuring continuous charging availability. Silicon carbide (SiC) switches are increasingly being used in power electronic converters, including IVSCs, due to their superior characteristics compared to traditional silicon (Si) switches [14], [15], [16]. In particular, SiC devices offer lower losses, higher operating frequencies, and improved thermal performance compared to traditional silicon-based devices. SiC-based IVSCs can achieve higher efficiency and power density, contributing to faster charging times and reduced energy losses [17], [18]. Indeed, the combination of lower losses and higher operating frequencies [19] in SiC-based IVSCs results in increased power density. By utilizing SiC switches, the IVSC can achieve higher power conversion efficiency and handle more power in a smaller physical footprint. This is particularly advantageous in applications where space is limited, such as in electric vehicles or renewable energy systems.

The design process of an IVSC encompasses multiple steps and considerations. It begins with defining specific requirements for the IVSC, including input voltage range, output voltage, power rating, efficiency targets, and other application-specific performance criteria. Next, it is crucial to select the most suitable number of interleaved modules based on both the application's requirements and constraints. This decision depends on factors such as desired power handling capacity, efficiency, reliability, and cost considerations. Increasing the number of modules enables higher power sharing and enhances fault tolerance. Furthermore, the selection of suitable power semiconductor devices, such as MOSFETs or IGBTs, depends on factors like desired switching frequency, voltage rating, current rating, and switching characteristics [20], [21], [22], [23], [24], [25].

In this paper, the interleaved traditional topology is investigated in relation to the dependency of converter efficiency on key design choices and on the constraints imposed by the application. The main constraints that characterize the analysis include the rated values of the DC-Link voltage and phase currents, as well as the desired output PWM frequency. Within this context, the selection of switching devices and of the number of interleaved legs defines the significant degrees of freedom in the design process. Since all the considered configurations satisfy the specified constraints, the optimal solution must be found by balancing the achieved efficiency and the complexity of the topology. Naturally, the design characterization using this approach strongly relies on the assigned set of constraints. Therefore, several cases can be considered that reflect typical values encountered in relevant applications, such as electric vehicles, renewable energy systems, and charging stations, among others. The main goal of the paper is to formulate a smart design procedure able to drive the configuration of the interleaved architecture towards an optimal choice with respect to the power module rating, the interleaved levels, the system efficiency, and the output current waveforms. For this purpose, the paper develops an approach to characterize through a straightforward procedure the dependence of the whole converter efficiency by the number of the deployed converters and other key design parameters.

In particular, converter losses are evaluated as a function of DC-Link voltage, switching frequency, and degree of interleaving. In this way, it is possible to easily identify the optimal areas to pursue during the detailed design phase to achieve the desired maximum efficiency.

The paper is organized as follows: after the introduction in section 1, the mathematical model of the interleaved converter topology is presented in section 2. In section 3, the design domain constraints are defined, and an investigation of losses in relation to key performance factors is presented. Besides, simulation results for several considered configurations are provided. Section 4 is devoted to the experimental validation of the proposed procedure. Eventually, conclusions are presented in section 5.

## 2 Mathematical model of an IVSC

Several key parameters have strong influence on the overall power losses of an IVSC such as the coupling methodology (inductors versus inter-phase transformers) and the modulation strategy. Since the paper mainly investigates the power losses dependence on the number of coupled converters, some design choices have been made in order to define the reference target. Indeed, IVSCs require the control of the recirculating currents through a proper exploitation of the mathematical model. Given that the control implementation, together with the modulation strategy, affects to a certain degree the system efficiency, it is important to formalize the used approach with the aim to outline the context on which both the numerical analysis and the experimental tests will be performed. In this section, the configuration under study is mathematically formalized by presenting the circuit configuration, the mathematical model and the modulation strategy.

**Figure 1:**
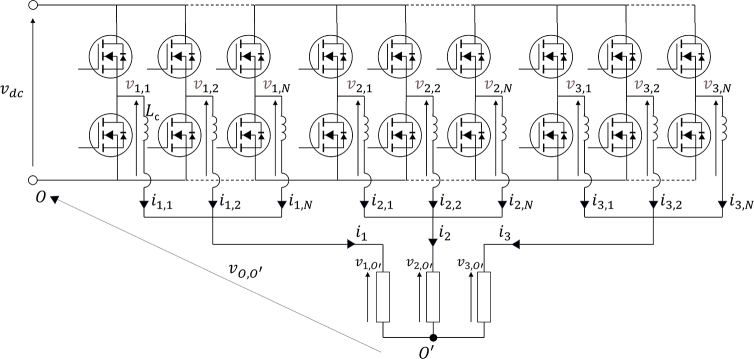
Configuration of the Interleaved Voltage Source Converter.

Fig. 1 shows the circuit configuration of an interleaved architecture built upon *N* identical three-phase converters. By applying the Voltage Kirchhoff Law to the closed circuits built upon the DC-Link, the generic *r*-th converter leg belonging to the *k*-th phase, and the corresponding load phase:

(1) $$v_{k,r}=L_{c}\frac{di_{k,r}}{dt}+v_{k,o^{\prime }}+v_{o^{\prime },o}\;\text{with}\;r\in \{1,...,N-1\}\;\text{and}\;k\in \{1,2,3\}$$

where $L_{c}$ is the recirculating inductance. Equation (1) is a system of 3×(N−1) simultaneous equations. Defining the common-mode $v_{k}^{0}$, $i_{k}^{0}$, and the differential-mode $v_{k,r}^{d}$, $i_{k,r}^{d}$ voltages and currents as below:

(2) $$v_{k,r}^{d}=v_{k,r}-v_{k}^{0}\;\;i_{k,r}^{d}=i_{k,r}-i_{k}^{0}\;\;v_{k}^{0}=\frac{1}{N}\sum_{r=1}^{N}v_{k,r}\;\;i_{k}^{0}=\frac{1}{N}\sum_{r=1}^{N}i_{k,r}$$

it yields:

(3) $$v_{k,r}=v_{k,r}^{d}+v_{k}^{0}\;\;i_{k,r}=i_{k,r}^{d}+i_{k}^{0}$$

Thus, by combining (3) and (1), the equations with respect to the differential and common mode components con be derived:

(4) $$v_{k,r}^{d}=L_{c}\frac{di_{k,r}^{d}}{dt}\;\;v_{k}^{0}=L_{c}\frac{di_{k}^{0}}{dt}+v_{k,o^{\prime }}+v_{o^{\prime },o}$$

It should be noted that when the electrical coupling is performed by means of inter-phase transformers, the inductance exposed by the converter to the common mode currents can be neglected in the second of (4). In this case, the load equations are reduced to:

(5) $$v_{k}^{0}=v_{k,o^{\prime }}+v_{o^{\prime },o}$$

Given the considered connection, the load is influenced only by the differential component $v_{k}^{\left(0,d\right)}$ of $v_{k}^{0}$:

(6) $$v_{k}^{\left(0,d\right)}=v_{k}^{0}-\frac{1}{3}\sum_{k=1}^{3}v_{k}^{0}$$

The first of (4) states that the differential currents are dependent only on the correspondent $v_{k,r}^{d}$ through the recirculating inductance $L_{c}$. Therefore, their control can be performed effectively by a properly sized Proportional-Integral (PI) regulator whose reference is set to zero. On the other hand, the load currents are dependent only on $v_{k}^{\left(0,d\right)}$. Consequently, the reference voltage $v_{k,r}^{⁎}$ of the generic converter leg can be expressed as:

(7) $$v_{k,r}^{⁎}=v_{k,r}^{d}+v_{k}^{\left(0,d\right)}+v^{\left(0,0\right)}$$

In (7) $v^{\left(0,0\right)}$ is the overall common mode component of the converters' legs output voltages. It does not influence either the differential currents, or the load currents. Consequently, it represents a freedom degree in the converter modulation. In this context, it is chosen through a min/max common mode injection:

(8) $$v_{0,0}=\frac{v_{dc}}{2}-\frac{1}{2}(v_{m}+v_{M})$$

where $v_{m}$ and $v_{M}$ are the minimum and maximum of the differential voltages set $v_{k,r}^{d}$, respectively. Finally, the modulation of the voltages references is performed through a phase shifting approach in order to increase both the equivalent modulation frequency and voltage levels by *N*.

## 3 Design of a highly efficient interleaved converter

In this section, the dependence of the converter efficiency on the number *N* of interleaved levels and on the employed switching devices is analyzed, by performing an assessment of the conduction and switching losses of the power modules that was accurate, but at the same time characterized by a not excessive computational effort. Since the converter losses depend also on other several parameters (power factor of the output current, DC-Link voltage level, modulation index, switching frequency, junctions' temperatures and so on), some assumptions have been made in order to keep reasonable the freedom degrees of the configurations while focusing on the variables which have a major impact on overall efficiency. In this context, two investigations have been performed: the first one addressed the dependence of the converter power losses on the DC-Link voltage $V_{\mathrm{dc}}$ and the output THD *k*, while keeping constant the output power factor $P_{f}$; the second one addressed the dependence of the converter power losses on the DC-Link voltage $V_{\mathrm{dc}}$ and the output power factor $P_{f}$, while keeping constant *k*. In each case, a set of surfaces describing the converter total losses' dependence on either $V_{\mathrm{dc}}$/*k* or $V_{\mathrm{dc}}$/$P_{f}$ is obtained. Each surface corresponds to a given converter configuration, i.e. both the number *N* of interleaved levels and the switching devices are assigned. Once the converter operating zone is defined, the built surfaces can be exploited by identifying the configuration which offers the best trade-off between performance and complexity.

**Figure 2:**
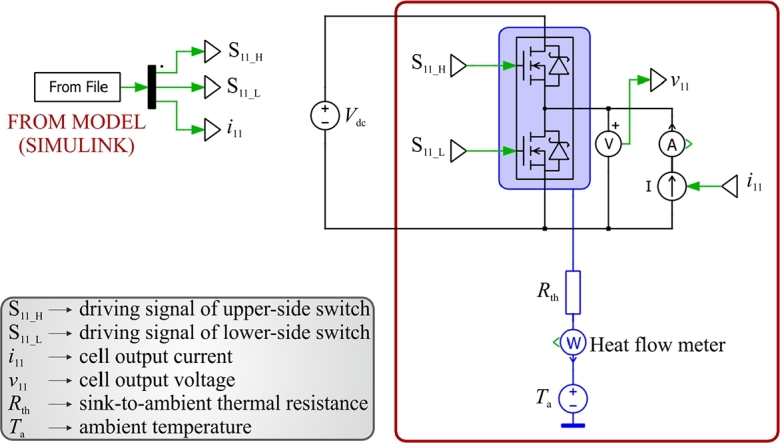
PLECS® thermal model of a generic cell of the interleaved converter.

**Figure 3:**
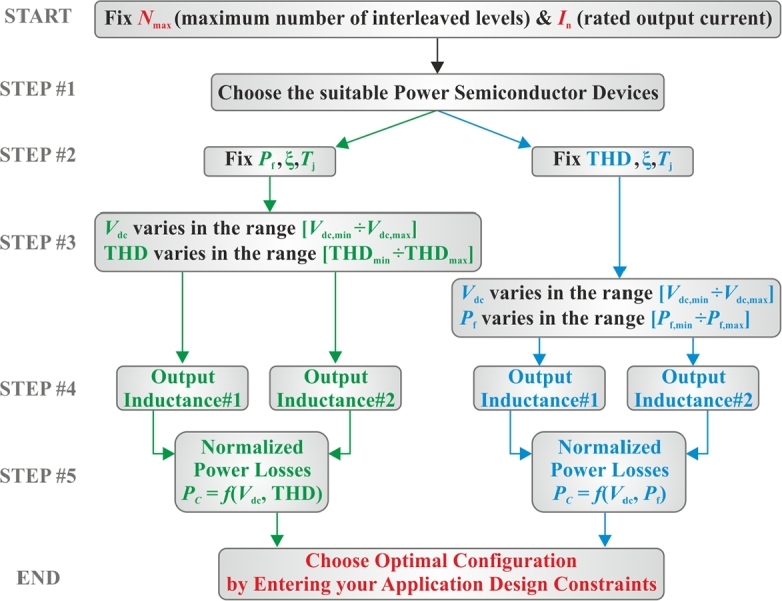
Flow-chart diagram of the proposed design procedure.

To obtain the target surfaces, the following method has been envisioned. Firstly, a set of simulations are preliminarily carried out in Matlab/Simulink environment, by implementing the control/modulation approach described in the previous section. With the aim to lighten the computational burden, the converter model is built upon the library of ideal switch components, which replaced in the simulation the power semiconductor devices. Secondly, the steady-state waveforms of voltage, current and control signals acquired through the Simulink model simulation are used as the input data of a PLECS® simulation, where the thermal models, provided by the manufacturer of the power semiconductor devices, are used to evaluate the conduction and switching losses. Fig. 2 shows the PLECS® configuration of a generic converter cell, while Fig. 3 reports a simplified flow-chart of the proposed multi-step design procedure. At the start point, the maximum number $N_{\max}$ of interleaved levels and the rated value of the converter output current must be selected, by considering the specific application under study. The first step is the choice of the suitable power semiconductor devices to be compared. Then, as previously explained, two paths must be investigated. At step 2, the first one (i.e., green path in Fig. 3) fixes the values of power factor $P_{f}$, modulation index *ξ*, and junction temperature $T_{j}$, while the second one (i.e., blue path in Fig. 3) fixes the values of THD, modulation index *ξ*, and junction temperature $T_{j}$. In particular, the operating maximum junction temperature is fixed to obtain a fair comparison in terms of thermal stress of the considered power semiconductor devices. At the third step, on both the paths the dc-link voltage $V_{\mathrm{dc}}$ varies from a minimum to a maximum value, which are defined on the basis of the considered specific application as well as of the limits for the THD, on the green path, and of the power factor, on the blue one. At step 4, two suitable values for the output inductance shall be selected with the aim of covering the possible load operating conditions. The next step (step 5) outcomes two surfaces representing the normalized power losses (i.e., normalized with respect to the case N=1 or rather not interleaved configuration) as function of the DC-Link voltage and THD, $P_{c}$($V_{\mathrm{dc}}$, THD), in the green case, and as function of the DC-Link voltage and power factor, $P_{c}$($V_{\mathrm{dc}}$, $P_{f}$), in the blue case. Finally, the obtained surfaces can be exploited by entering the design constraints of the specific application under study, thus leading to the identification of the optimal solution which, as already stated, represents a compromise between desired performance and topology complexity.

As an example of application of the proposed methodology, the three half-bridge power modules of Table 1 were considered, based on SiC technology and chosen from ROHM® catalog.

**Table 1:** Considered power modules from ROHM® catalog.

Module ID	Drain current (A)	Drain-Source voltage (V)
BSM120D12P2C005	134	1200
BSM180D12P3C007	180	1200
BSM400D12P3G002	358	1200

The rated value $I_{n}$ of the output current was set to 300 A, while the interleaved level *N* was capped at 3. Given these constraints, for a three-phase inverter, the six possible configurations of Table 2 can be considered. Naturally, because the proposed methodology introduces some assumptions, it is mandatory to validate, through experimental tests, the numerically computed losses with respect to a significant set of operating points. The validation will be presented in section 4.

**Table 2:** Possible configurations for 300 A rated current.

Module ID	Total number of modules	*N*
BSM120D12P2C005	9	3
BSM180D12P3C007	9	3
BSM400D12P3G002	9	3
BSM180D12P3C007	6	2
BSM400D12P3G002	6	2
BSM400D12P3G002	3	1

### 3.1 Dependence on DC-link voltage and output THD

**Figure 4:**
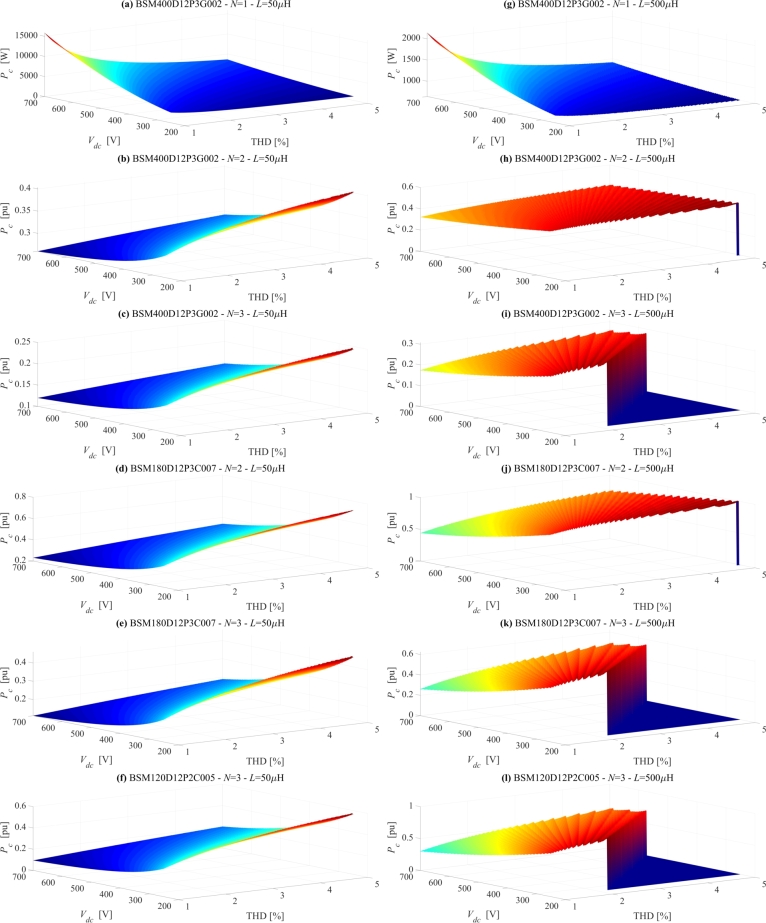
Converter's total power losses for *V*_*dc*_ and THD variations, assuming *L* = 50 μH (subplots a-f) and *L* = 500 μH (subplots g-l). Subplots (a) and (g) depict the converter losses for the not interleaved configuration, i.e. *N* = 1, while the other subplots show the relative losses of the other configurations with respect to the not interleaved one.

A first set of results is derived by imposing the power factor ($P_{f}=0.9$), the modulation index (*ξ* = 0.95) and the junctions' temperatures of all power modules ($T_{j}$ = 70 °C). The computation of the converter losses is then performed by varying the DC-Link voltage $V_{\mathrm{dc}}$ in the interval [250 V, 700 V] and the output THD *k* in the interval [1%, 5%]. A simplified expression of *k* can be derived by assuming the output voltages as obtained from the superimposition of the modulating signals, which at steady state are purely sinusoidal, and of the square waves, which represent the effect of the PWM, having an amplitude equal to $V_{\mathrm{dc}}/(3N)$ and a frequency equal to $Nf_{\mathrm{sw}}$, with $f_{\mathrm{sw}}$ switching frequency of the power modules. Once the converter output voltages are formalized in this way, with reference to an inductive symmetrical three-phase load, the currents residual harmonics assume a triangular behavior. This approach leads to the following approximated formula of *k*:

(9) $$k=100\frac{V_{dc}}{12\sqrt{2}LI_{n}N^{2}f_{\mathrm{sw}}}$$

where *L* is the output phase inductance. The dependence of *k* on the reciprocal of the square of the interleaved levels *N* is established by the phase shifted modulation of the *N* converters, which increases both the output modulation frequency and the output voltage resolution. Fig. 4 shows the results for *L* = 50 μH (a-f) and for *L* = 500 μH (g-l). Subplots (a) and (g) depict the converter losses for the not interleaved configuration, N=1, while the other subplots show the relative losses of the other configurations with respect to the not interleaved one. The behaviors depicted in Figs. 4.a and 4.a show that, as expected, the total converter power losses $P_{c}$ increase with $V_{dc}$ and decrease with *k*. It can be noted that the partial derivative of $P_{c}$ with respect to $V_{dc}$ increases with lower values of *k*, while the partial derivative of $P_{c}$ with respect to *k* increase with higher values of *k*. This functional dependence leads to a high ratio between the maximum and the minimum value for $P_{c}$: around 5 for *L* = 50 μH versus about 2.5 for *L* = 500 μH. On the other hand, the variation of the ratio with the inductor value causes the losses' dependence on the *L* value to become gradually smoother for higher values of *k* and lower values of $V_{dc}$. The relative power losses behaviors depicted in Figs. 4.b-4.f highlight that, while the losses' reduction granted by the interleaved configurations built upon the reference power module is weakly dependent on $V_{dc}$ and *k*, the exploitation of the reduced requirement for the current rating through the deployment of lower class power module introduces a strong dependence of the relative power losses on $V_{dc}$ and *k*. In particular, the interleaved configurations built upon lower class power modules give rise to a significant power losses reduction at high values of $V_{dc}$ and low values of *k*, where, on the other hand, the absolute power losses of the reference not interleaved configuration reach their maximum value. Eventually, Figs. 4.h-4.l show qualitative behaviors similar to the previous ones, with two main differences. Firstly, the power losses reduction is significantly lower, i.e., the exploitation of the interleaved configuration appears less effective. Secondly, some operating points (flat red area in the respective surfaces) would lead to a too low switching frequency, resulting in a minimum sampling time (1 ms) which is not compatible with an acceptable control's frequency bandwidth.

### 3.2 Dependence on DC-link voltage and power factor

**Figure 5:**
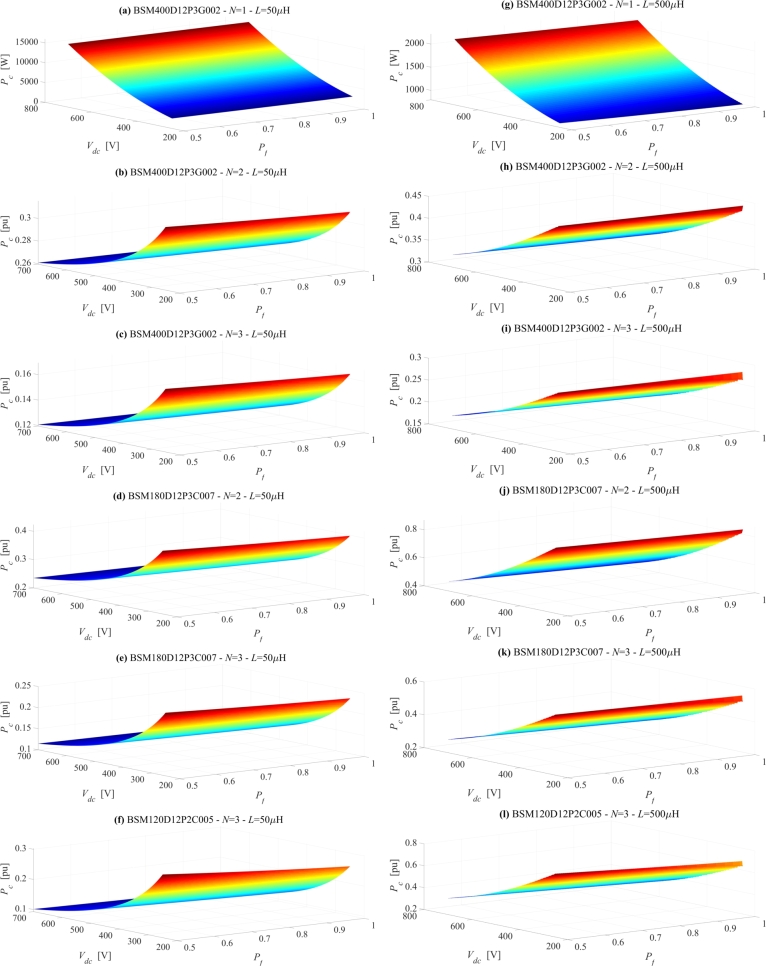
Converter's total power losses for *V*_*dc*_ and *P*_*f*_ variations. Assuming *L* = 50 μH (subplots a-f) and *L* = 500 μH (subplots g-l). Subplots (a) and (g) depict the converter losses for the not interleaved configuration, i.e. *N* = 1, while the other subplots show the relative losses of the other configurations with respect to the not interleaved one.

A second set of results is derived by imposing the output THD (*k* = 1%), the modulation index (*ξ* = 0.95) and the junctions' temperatures of all power modules ($T_{j}$ = 70 °C). The computation of the converter losses is then performed by varying the DC-link voltage in the interval [250 V, 700 V] and the power factor $P_{f}$ in the interval [0.5, 1]. Fig. 5 shows the results for *L* = 50 μH (a-f) and *L* = 500 μH (g-l). Again, the power losses of the interleaved configurations are normalized with respect to the ones of the not interleaved configurations, depicted in the subplots (a) and (g). From the represented behaviors, it can be observed that both the absolute power losses and the relative ones depend poorly on the power factor. The result is expected, since a power factor variation modifies substantially the current sharing between the switching device and the recirculating diode, which exhibit similar resistances. It can be therefore concluded that the optimization procedure can be performed with respect to the rated values without significantly loosing accuracy.

## 4 Experimental validation

**Figure 6:**
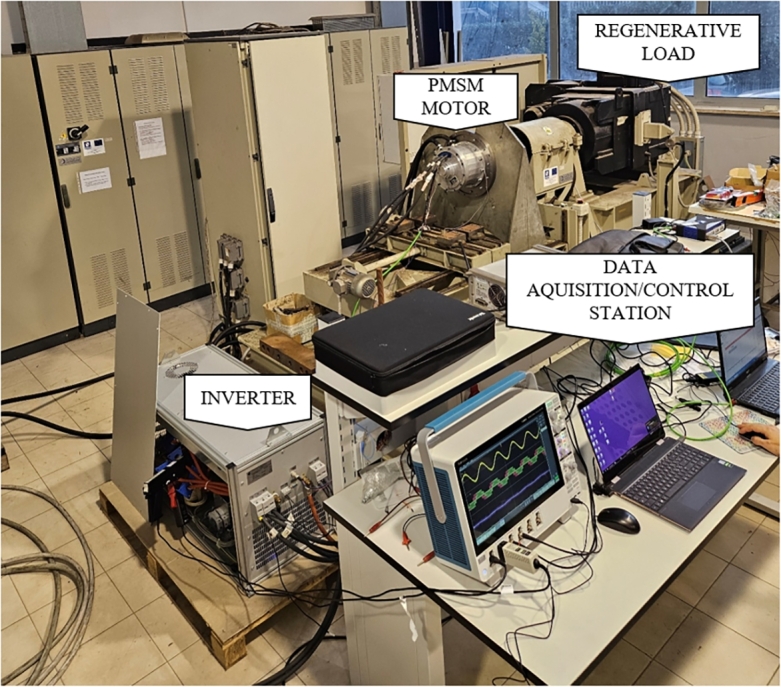
Experimental set up.

Since it is difficult to experimentally reproduce the whole data sets proposed in section 3, the experimental validation has been performed on one considered meaningful case study, i.e. the 2-levels interleaved configuration built upon the BSM400D12P3G002 modules. The converter has been tested in an electric drive system, see Fig. 6, whose main characteristics have been reported in Table 3.

**Table 3:** Main data of the experimental setup.

Main	Switching	Rated	Rated	Synchronous	Poles	DC-Link
parameters	frequency	power	speed	inductance	pairs	voltage
Values	10 kHz	85 kW	5800 rpm	63 μH	5	270 V

It should be noted that the DC-Link voltage (270 V) is in the considered numerical range [250 V, 700 V], while the synchronous inductance (63 μH) is very close to one of the contemplated values (50 μH). Given that the DC-Link voltage is constrained, a comparison with the numerical results would have involved either the output current THD variation at fixed power factor or the load power factor variation at fixed THD.

**Figure 7:**
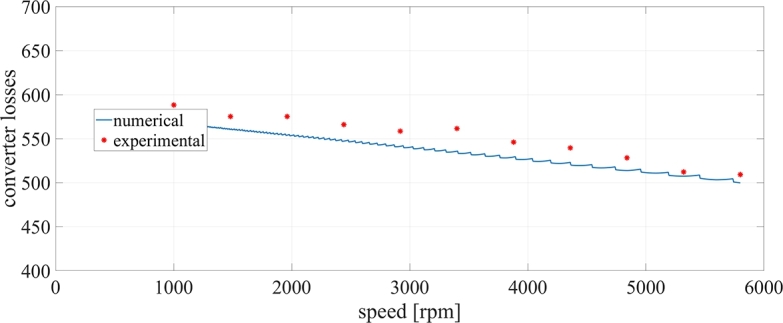
Comparison of the experimental measured converter losses (red dots) with the numerical ones (blue line).

**Figure 8:**
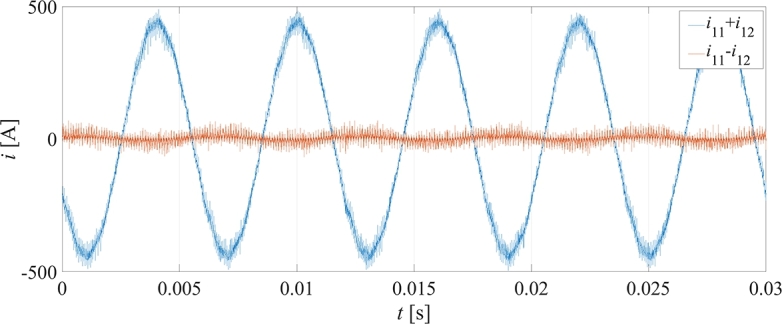
Output and recirculating currents for the first converter phase at 2000 rpm.

Nonetheless, it is acceptable to validate the proposed approach through other available means once an extensive set of comparisons is performed. In this context, the tests have been executed by varying the speed of the PMSM motor in the range [1000 rpm, 5800 rpm] while keeping the output currents RMS at 300 A. In the considered operating conditions both the THD and the power factor are subjects to weak variations, while the modulation index varies linearly with the speed. With the same approach exploited to build up the converter losses surfaces, the numerical behavior of the converter losses as a function of the motor speed is obtained. For each experimental operating point, the output currents of the converter obtained in Simulink environment are used as inputs of a PLECS® simulation, which outputs the overall converter power losses. Fig. 7 shows the comparison of the numerically computed overall converter power losses (blue line) with the measured ones (red dots). From the comparison it can be deduced that the measured converter losses are very close to numerical ones in the whole speed range. The difference is under the 8%, which, considering the absolute value of the calculated losses, confirms the goodness of the proposed approach. On the other hand, given that the measured values are systematically above the numerical ones, it can be concluded that the converter losses are underestimated. This deviation is substantially caused by the recirculating currents, which, on the top of the expected ripple driven by the modulation frequency, are characterized also by an undesired oscillation at the fundamental output frequency, as can be seen in Fig. 8. This additional ripple component, which is caused by a small difference between the current transducers gains, leads to an increase of the overall RMS of the converter currents with a consequent increase of the system losses. It is worth noting that, for the considered application targeted to an electrical drive, while the converter overall power losses vary weakly with the speed, the output power, given that the currents' RMS is kept constant, vary almost linearly with the speed. Consequently, the converter overall efficiency also depends significantly on the speed. For the considered system, the converter overall efficiency reaches its maximum value at 5800 RPM (around 99%) and its minimum value at 1000 RPM (around 0%).

## 5 Conclusion

In the paper a smart design procedure for IVSC has been proposed, which is able to drive the configuration of the interleaved architecture towards an optimal choice of the power module rating, the number of interleaved levels, the system efficiency, and the output current waveforms. The proposed procedure is based on the determination of surfaces representing the normalized trend behavior of conduction and switching losses of the power semiconductor devices, which mainly depend on the number *N* of interleaved levels and on the characteristics of the chosen power semiconductor devices. Only the parameters which have a major impact on the losses have been taken into account: power factor of the output current, DC-Link voltage level, THD, modulation index, switching frequency, and junction temperature. By fixing and/or varying these parameters along with the load conditions, it is possible to simply determine normalized power losses surfaces, which can be exploited by considering the design constraints of the specific application. This leads to the possibility of choosing the corresponding optimal configuration. In particular, in the paper the proposed methodology has been applied by considering real silicon carbide devices, currently available on the market, with different values of rated current. The experimental results obtained testing a real IVSC used in a real electric drive system, confirm the validity of the results obtained by means of the proposed approach.

## CRediT authorship contribution statement

**Adolfo Dannier:** Conceptualization, Data curation, Formal analysis, Methodology, Writing – review & editing. **Gianluca Brando:** Data curation, Investigation, Methodology, Software, Writing – review & editing. **Marino Coppola:** Conceptualization, Data curation, Investigation, Writing – original draft. **Ciro Attaianese:** Conceptualization, Supervision, Validation, Writing – review & editing.

## Declaration of Competing Interest

The authors declare the following financial interests/personal relationships which may be considered as potential competing interests: Adolfo Dannier reports article publishing charges was provided by 10.13039/100007195University of Naples Federico II. If there are other authors, they declare that they have no known competing financial interests or personal relationships that could have appeared to influence the work reported in this paper.
